# Supplementary material for: Amorphization-Mediated Si‑I to Si‑V Phase Transition and Reversible Amorphous Si‑V Phase Memory in Silicon Nanoparticles
Source: Nano Lett. 2026 Sep 15;26(38):12702–8. doi: 10.1021/acs.nanolett.6c02592 (PMC13632584; doi:10.1021/acs.nanolett.6c02592)
Supplement: Supplementary file 1 [file nl6c02592_si_001.pdf]

Supplementary Information

Amorphization-Mediated Si-I to Si-V Phase Transition and Reversible Amorphous–Si-V Phase Memory in Silicon Nanoparticles

Ziye Deng[1], Reza Namakian[1], and Wei Gao[1,2]

[1]J. Mike Walker ′66 Department of Mechanical Engineering, Texas A&M University, College Station, Texas 77843, United States
[2]Department of Materials Science & Engineering, Texas A&M University, College Station, Texas 77843, United States

Supplementary Note 1
Phase Transition of 10 nm Si Particle Under Triaxial Compression Predicted by SW and Tersoff Potentials

Previous molecular dynamics studies employing empirical force fields have primarily investigated the uniaxial loading of Si nanoparticles in the 10nm class. Using the SW potential, Chrobak et al. [S1] modeled a 5.2nm-radius particle and found a dislocation seed on $(\bar{1}01)$ plane while the larger nanoparticles form perfect dislocations. In contrast, simulations using the Tersoff potential have yielded different outcomes. Valentini et al. [S6] reported that uniaxial compression of 10nm particles leads to an amorphous skin that crystallizes into tetragonal Si-II. Subsequent work by Hong et al. [S3], also using the Tersoff potential, further confirmed the formation of these intermediate tetragonal metastable phases which gradually transformed back to the original Si-I phase during the unloading process. The metastable is concluded as deconfinement effect from the Si-I transit to perfect Si-II. These divergent observations underscore the sensitivity of the predicted mechanism to both the chosen force field and the degree of hydrostatic confinement.

To enable a direct comparison, SW, Tersoff (1989), and GAP-18 simulations were compared under identical triaxial compression conditions. Under the triaxial loading condition, the simulation based on SW predicts a combination of dislocation-mediated plasticity and phase transformation. The initial onset of plasticity occurs at approximately 10% strain (refer to Figure 1B in the main text) with the nucleation of dislocation embryos. These embryos subsequently expand into perfect dislocation loops with a Burgers vector of $\mathbf{b} = \frac{1}{2}\langle 110\rangle$ as depicted in Fig. S1. This dislocation-mediated response is consistent with that reported in previous uniaxial compression studies [S1]. With continued compression, the nearby Si-I lattice becomes increasingly distorted, which in turn disrupts the integrity of these perfect dislocations. Concurrently, the triaxial loading causes the spherical shape to undergo a morphological evolution toward the nanocube-like geometry. Throughout this process, the core of the nanoparticle maintains its Si-I structure (Fig. S2). Upon further compression to 14% strain, a phase transformation is initiated. A portion of the atoms transitions from the four-coordinated Si-I structure to a higher-density phase characterized by a coordination

number greater than four. This new structure is identified as the body-centered tetragonal (BCT5) phase, a conclusion corroborated by the radial distribution function (RDF) presented in Fig. S3.

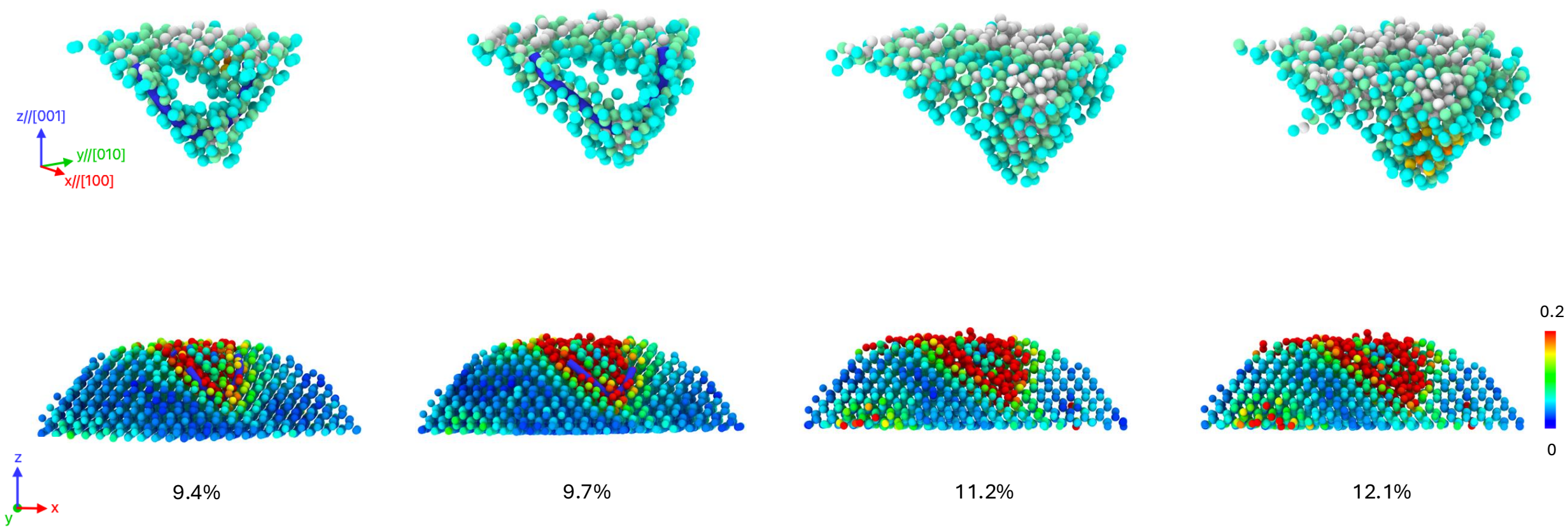

Figure S1: Atomic configurations illustrating dislocation evolution based on SW. Atoms are colored by the 'Identify Diamond Structure' modifier [S4], where perfect cubic diamond atoms are deleted for clarity, and local shear strain, measured by the 'Atomic Strain' modifier. Perfect dislocations are identified using the Dislocation Extraction Algorithm (DXA).

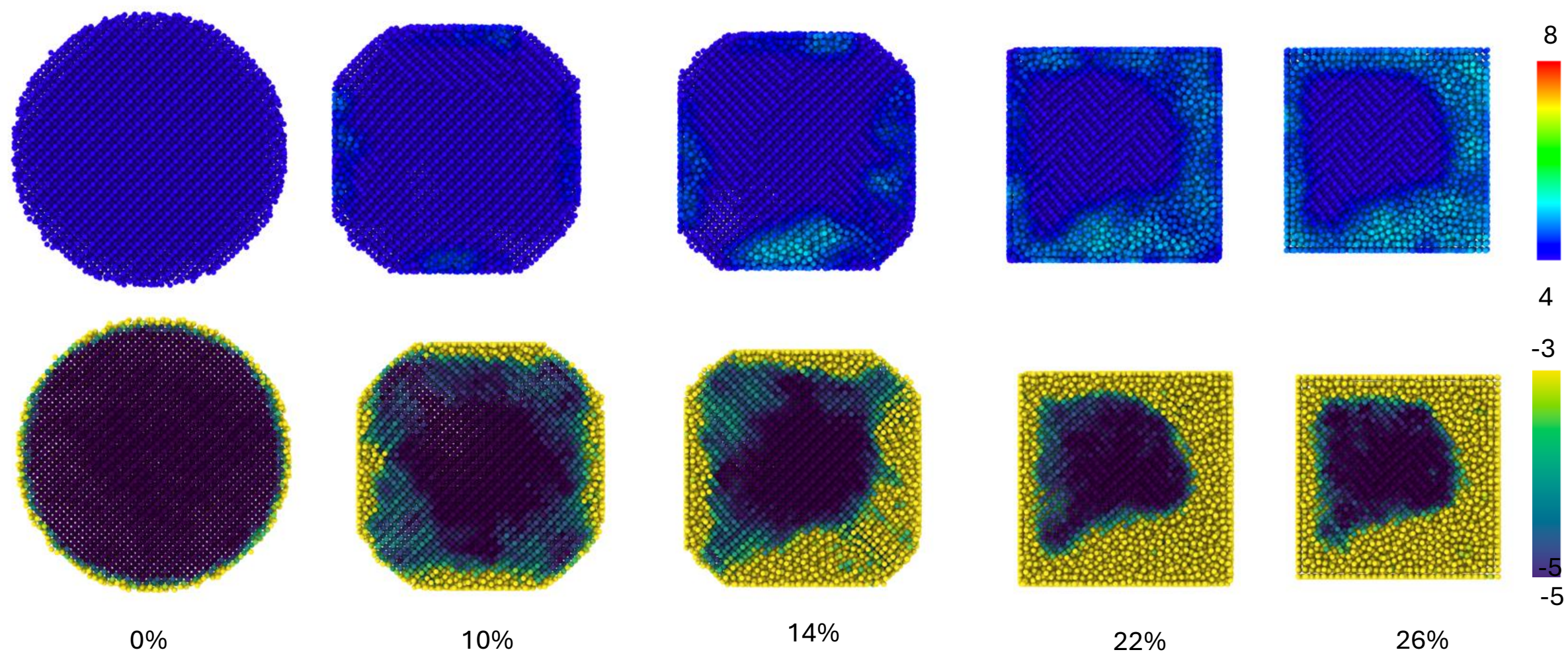

Figure S2: Cross sectional snapshots of a 10 nm Si nanoparticle under triaxial compression by SW, shown as a function of engineering strain (columns). Atoms are colored by coordination number(upper) and order/disorder descriptor (lower). The coordination number is calculated with a cutoff distance of 2.85 Å and averaged over a 3 Å radius. The order/disorder descriptor is averaged over a 3 Å radius.

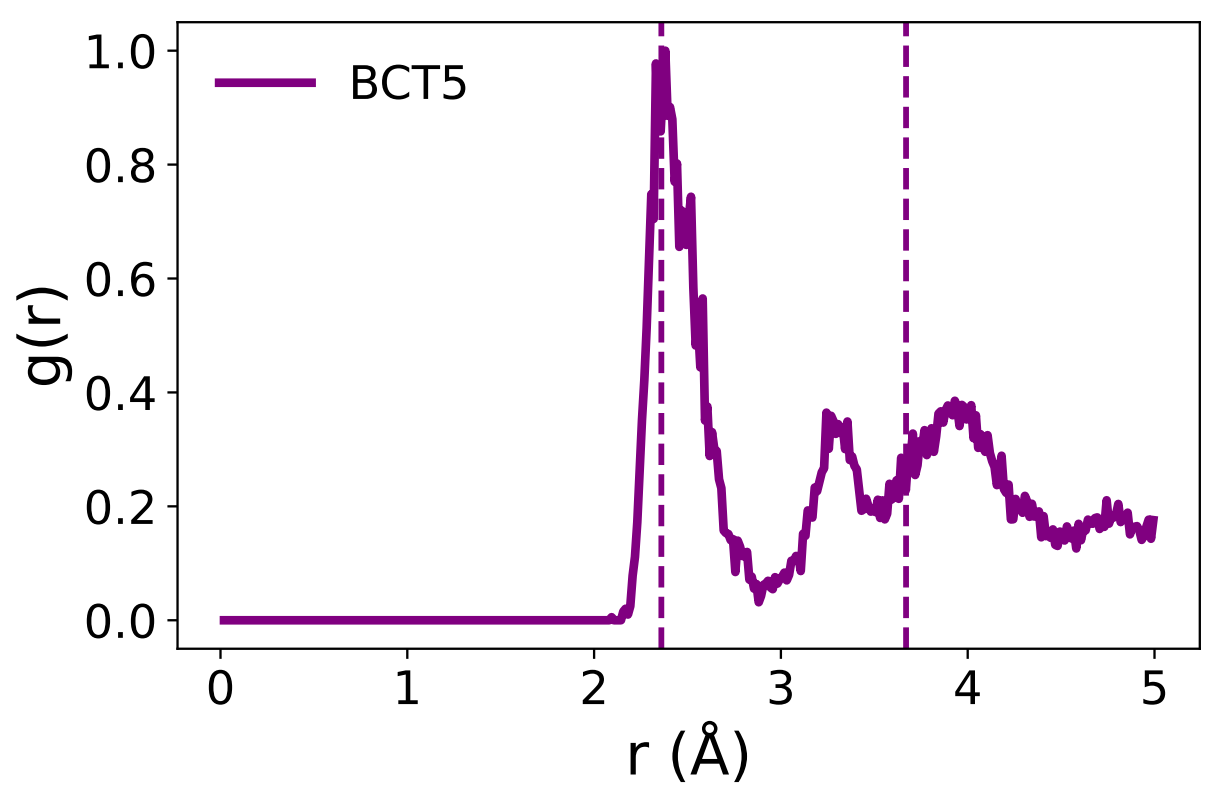

Figure S3: RDF for BCT5 phase with dashed lines marking characteristic peak positions of the corresponding ideal phases.

In contrast, the simulation using Tersoff predicts a crystal to crystal phase transformation at around 18% strain, qualitatively similar to the metastable transformations reported in previous uniaxial compression studies [S3]. The resulting transformed structure is identified as a metastable phase, as it reverts to the original Si-I during the unloading process. Although the reverse transformation is not detailed here, the term "metastable phase" is used for clarity. This new phase is characterized by a high coordination number (CN) approaching 12. As shown by the RDF in Fig. S5, this transition involves a significant contraction of the second-nearest neighbor distance from 3.84 Å in the initial Si-I structure to approximately 3.3 Å in the metastable phase. A detailed visualization of this structural evolution is provided in the data repository.

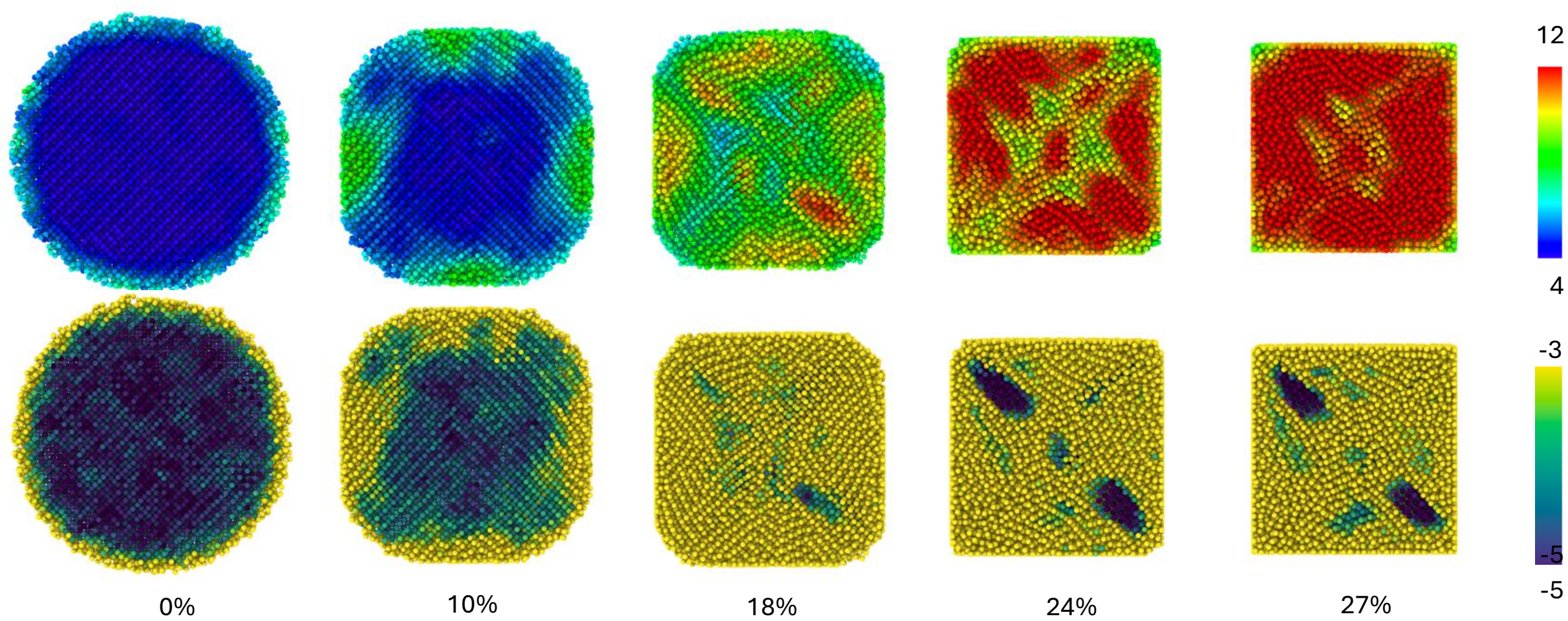

Figure S4: Cross sectional snapshots of a 10 nm Si nanoparticle under triaxial compression by Tersoff, shown as a function of engineering strain (columns). Atoms are colored according to their coordination number(upper) and order/disorder descriptor(lower). Both quantities are spatially averaged over a 3 Å radius; the CN calculation uses a cutoff distance of 3.5 Å.

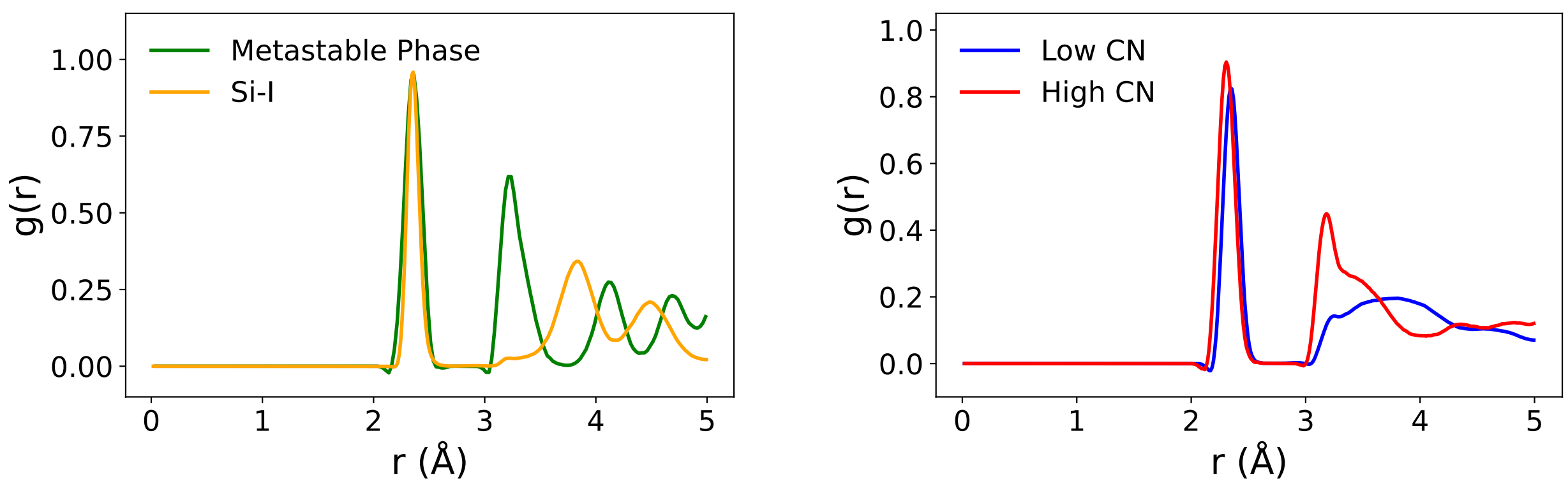

Figure S5: RDF for Si-I, the intermediate phase, the metastable phase and the distorted metastable phase. (a) RDFs of the Si-I phase and the metastable phase. (b) RDFs of low order/disorder descriptor regions, classified by lower coordination number$\approx$ 8(intermediate phase) and CN $\approx$ 12 (distorted metastable phase).

To elucidate the phase transformation pathway, we analyzed the local coordination number and order/disorder descriptor, as depicted in Fig. S4. Following the initial elastic compression, an intermediate phase with a CN of approximately 6 nucleates in the regions near the indenters and subsequently propagates inward. The RDF of this intermediate structure (Fig. S5) possesses short-range order but lacks long-range periodicity. Upon further compression, the high-density metastable phase nucleates from this intermediate structure as shown in GitHub repository. However, the transformation is not uniform throughout the configuration. Regions with a high coordination number similar to the metastable phase are also observed. The RDF of these regions reveals a structural signature similar to the ordered metastable phase, notably a prominent second-neighbor peak near 3.3 Å, suggesting they are a distorted variant of the crystalline metastable structure.

Supplementary Note 2
Structural Analysis of Si-I→HDA→VHDA→Si-V during Initial Loading

To elucidate the amorphization and recrystallization process during the initial loading of the Si-I nanoparticle, we performed a detailed analysis using order/disorder descriptor, coordination number, RDF and angular distribution function (ADF). The order/disorder descriptor (Fig.S6(a)) separates crystalline from disordered regions, while the coordination number, RDF and ADF are used to identify specific crystalline and amorphous states. At early strain (15% to 25%), the coordination number (Fig.S6(b))shows an abrupt change from 4 to approximately 6 as Si-I becomes amorphous. The disordered phase exhibits characteristics of high-density amorphous silicon (HDA) (Fig.S6(c) and (d)) and differs significantly from the low-density amorphous (LDA). The RDF first minimum is noticeably above zero, whereas in LDA it approaches zero. In addition, the ADF exhibits a more noticeable peak around 55°, whereas LDA shows a sharp peak at around 109° with a much smaller peak around 55°. Clearly, the coordination number, RDF and ADF collectively indicate the formation of HDA silicon.

Next, we present the evidence that very-high-density amorphous (VHDA) serves as the precursor of Si-V during initial loading. At ∼28% strain, the coordination number in the disordered shell

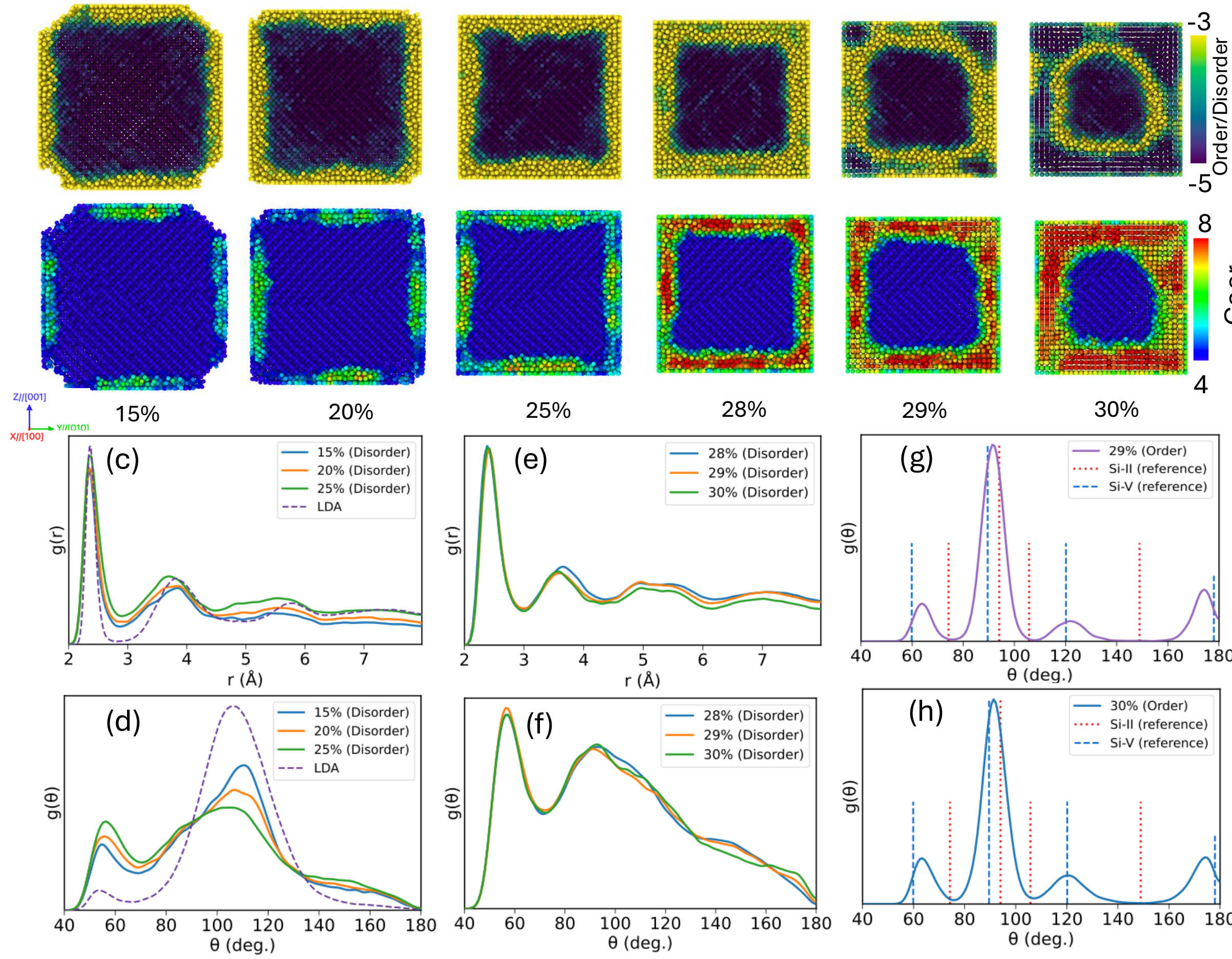

Figure S6: (a) Cross-sectional snapshots of a 10 nm Si nanoparticle, colored by order/disorder descriptor. (b) Coordination number map. (c) RDF of disordered region(15% to 25%). (d) ADF of disordered region (15% to 25%). (e) RDF of disordered region(28% to 30%). (f) ADF of disordered region (28% to 30%). (g) ADF of new ordered region (29%). (h) ADF of new ordered region(30%) compared to Si-II reference (dotted line) and Si-V reference(dashed line).

increases to approximately 8. The RDF and ADF for the disordered regions from 28% to 30% (Fig. S6(e) and (f)), also show VHDA signatures: the RDF develops a moderate peak around 5Å(absent in HDA), and ADF shows a pronounced peak around 55°. We provide additional evidence that the newly formed crystalline phase is Si-V. The ADFs of the newly nucleated ordered regions at 29% and 30% strains (Fig.S6(g,h)) agree closely with the Si-V reference and clearly deviate from the Si-II reference. Together, these analyses support the assignment of a Si-I→HDA/VHDA-like a-Si→Si-V pathway during the first loading cycle, with no evidence of an intermediate Si-II-like ordered state in the analyzed configurations.

To further validate the Si-V assignment and examine whether the amorphous-mediated Si-V nucleation observed during continuous loading is merely a transient consequence of the high MD loading rate, we performed a fixed-indenter relaxation test. The configuration was selected from the first loading trajectory at approximately 27% engineering strain, immediately before the stress plateau and rapid growth of the high-pressure phase. The nanoparticle was then relaxed at 300 K while all six indenter positions were held fixed. Because no additional macroscopic strain

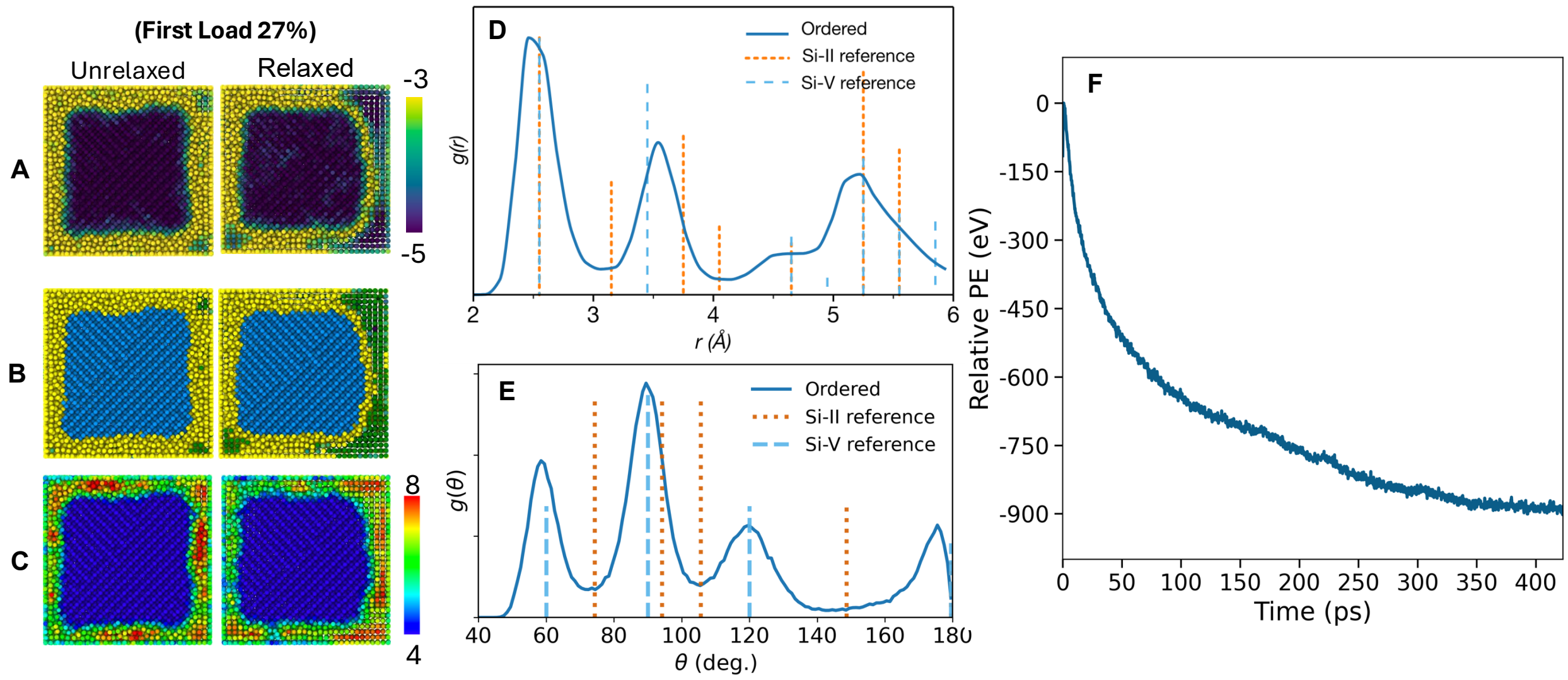

Figure S7: Fixed-indenter relaxation analysis of the configuration selected from the GAP loading trajectory at approximately 27% engineering strain. (**A**) Cross-sectional snapshots colored by order/disorder descriptor before and after fixed-indenter relaxation. (**B**) Corresponding phase maps. (**C**) Coordination number maps before and after relaxation. (**D**) RDF of the new ordered atoms formed compared with Si-II and Si-V reference peak positions. (**E**) ADF of the newly formed ordered atoms compared with Si-II and Si-V reference peak positions. (**F**) Relative potential energy during fixed-indenter relaxation.

was imposed during this test, the structural evolution reflects relaxation under the same triaxial confinement rather than continued compression.

As shown in Fig. S7(A–C), the unrelaxed configuration consists of a Si-I core surrounded by a high-density amorphous shell. The coordination-number map shows that this shell contains HDA/VHDA-like local environments, with coordination increasing from the fourfold Si-I core toward six- to eightfold coordination near the compressed surface and corner regions. After fixed-indenter relaxation, the same core–shell topology is preserved: the amorphous shell is not eliminated and does not reconstruct back into Si-I. Instead, part of the high-density amorphous shell becomes ordered while the indenter positions remain fixed.

The RDF and ADF of the newly ordered atoms formed during relaxation, shown in Fig. S7(D,E), exhibit peak positions that are more consistent with the Si-V reference than with the Si-II reference. This supports the assignment of the relaxed ordered domains as Si-V-like nuclei rather than as a Si-II intermediate. The relative potential energy decreases during relaxation and approaches a plateau, indicating that the resulting configuration is a relaxed metastable state under fixed triaxial confinement (Fig. S7(F)). Therefore, the amorphous shell is not simply a short-lived loading-rate effect that disappears upon relaxation; rather, it acts as a metastable HDA/VHDA-like precursor from which Si-V can nucleate. Together with the continuous-loading RDF, ADF, and coordination-number analyses, this fixed-indenter relaxation test supports the Si-I→HDA/VHDA-like a-Si→Si-V pathway during the first loading cycle.

We note that this fixed-indenter relaxation test does not replace a full quasistatic loading-rate study. Instead, it provides a local structural-relaxation check at the critical configuration near the onset of Si-V nucleation. The persistence of the amorphous shell and the formation of Si-V domains during fixed-indenter relaxation indicate that the amorphous-mediated pathway is not

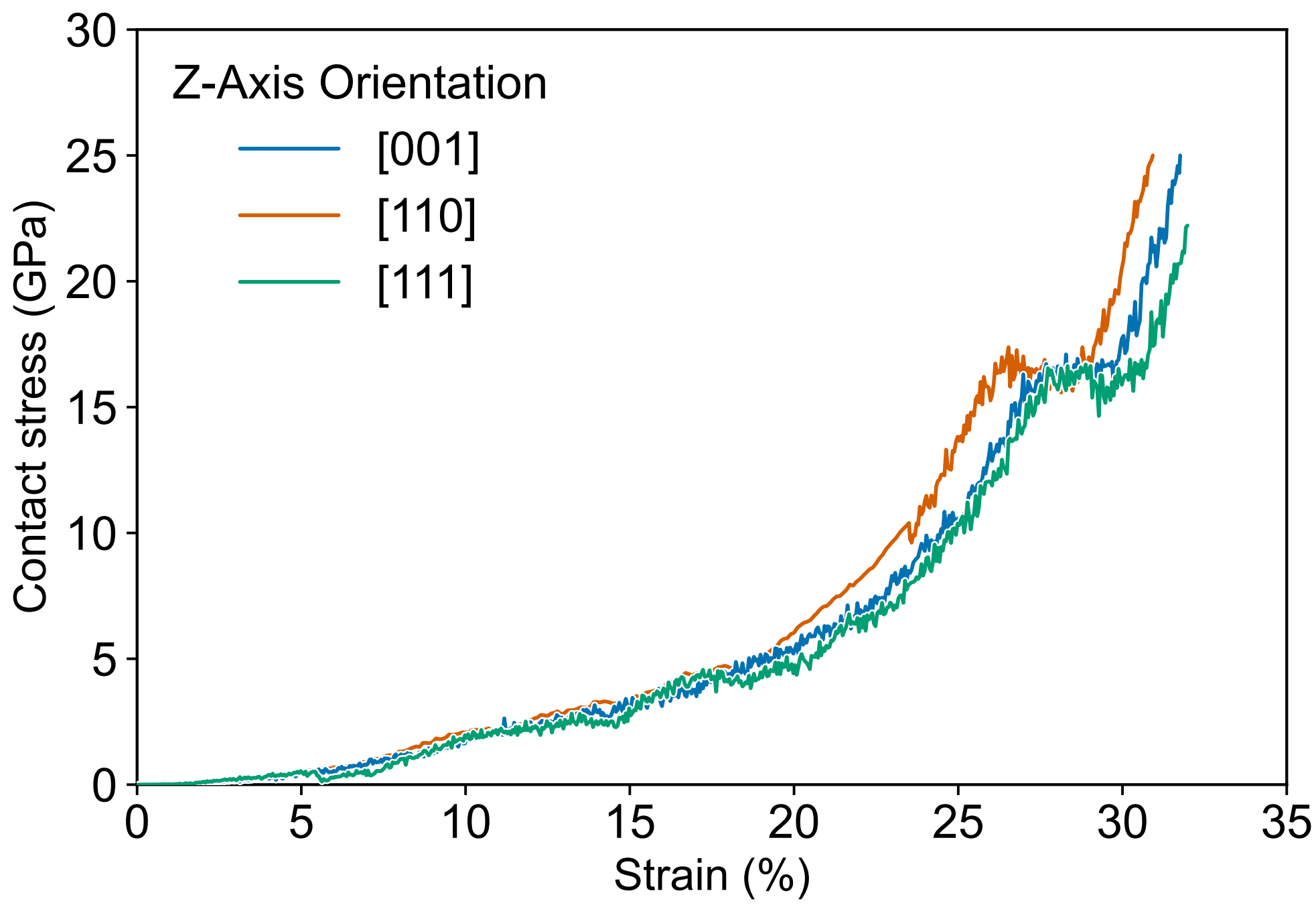

Figure S8: Contact stress–strain curves obtained from MD simulations of 10-nm Si nanoparticles with their z axes aligned with the [001], [110] and [111] crystallographic directions.

solely a transient consequence of loading motion.

Supplementary Note 3
Simulations for Nanoparticles with Different Crystallographic Orientations

The powder XRD signal in the experiment averages over nanoparticles with different crystallographic orientations and contact environments. We therefore varied the particle orientation as one controlled source of particle-level heterogeneity, while keeping the loading protocol unchanged. The particle with its z axis aligned with [001] was the baseline case used in the main study. Two additional 10-nm particles, with their z axes aligned with [110] and [111], were independently constructed using Atomsk. For each case, an oriented diamond-cubic crystal was first generated using the axes listed in Table S1, and a spherical particle was then carved from the oriented crystal. All three particles were subjected to the same annealing, cooling, and six-indenter loading protocol.

Table S1: Crystallographic orientations of the three independently constructed Si nanoparticles relative to the x-, y-, and z-axes.

**Case**	**x-axis**	**y-axis**	**z-axis**
1	[100]	[010]	[001]
2	$[\bar{1}10]$	[001]	[110]
3	$[1\bar{1}0]$	$[11\bar{2}]$	[111]

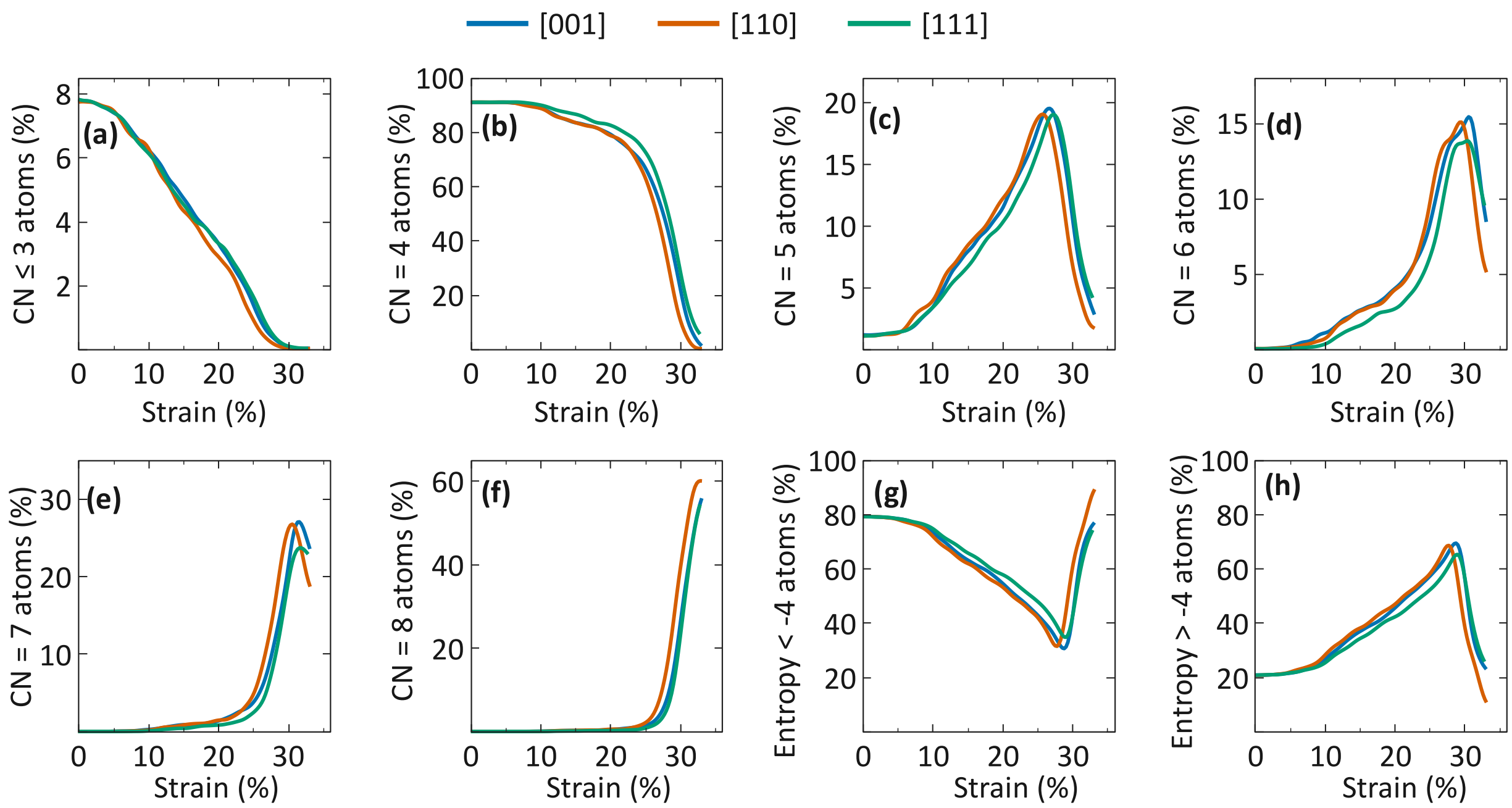

Figure S9: Global coordination-number and order/disorder descriptor populations as functions of strain for the three particle orientations. Panels (a–f) show the coordination-number populations calculated over all atoms in each particle using a 2.85 Å neighbor cutoff. Panels (g) and (h) show the order/disorder descriptor populations, respectively.

The contact stress–strain curves remain close at small strains and begin to separate beyond approximately 20% strain (Fig. S8). The onset strain of the transformation-associated plateau increases in the order [110], [001] and [111]. Despite these onset shifts, all three curves exhibit the same qualitative mechanical sequence: a transformation-associated plateau followed by subsequent hardening. Thus, crystallographic orientation primarily shifts the onset of the transformation-associated response rather than changing the mechanical evolution. The coordination-number populations show similar trends for all three orientations (Fig. S9). The CN= 4 population decreases with compression; the CN= 5 and CN= 6 populations first increase and then decline; and the CN= 7 and CN= 8 populations increase at larger compression. The order/disorder descriptor populations also follow similar overall trends. Quantitative differences at a given strain are consistent with the orientation-dependent onset shifts, particularly the earlier entry of the [110] case into the transformation-associated regime. Equal strain therefore does not necessarily correspond to an equivalent mechanical or structural state across orientations. The central cross sections in Figs. S10–S11 show the strain-resolved evolution for the two additional orientations. In each case, disordered material first develops near the particle surface and then extends inward. At larger compression, Si-V regions nucleate within the densified disordered material. Because the transformation onset depends on orientation, comparisons at equal strain can correspond to different stages of the mechanical response. We therefore also compared the three orientations at contact stresses of approximately 10, 18, and 28 GPa (Fig. S12). This stress-matched comparison shows a common outward-to-inward progression of the disordered region for all three orientations, followed by the development of ordered regions at larger compression.

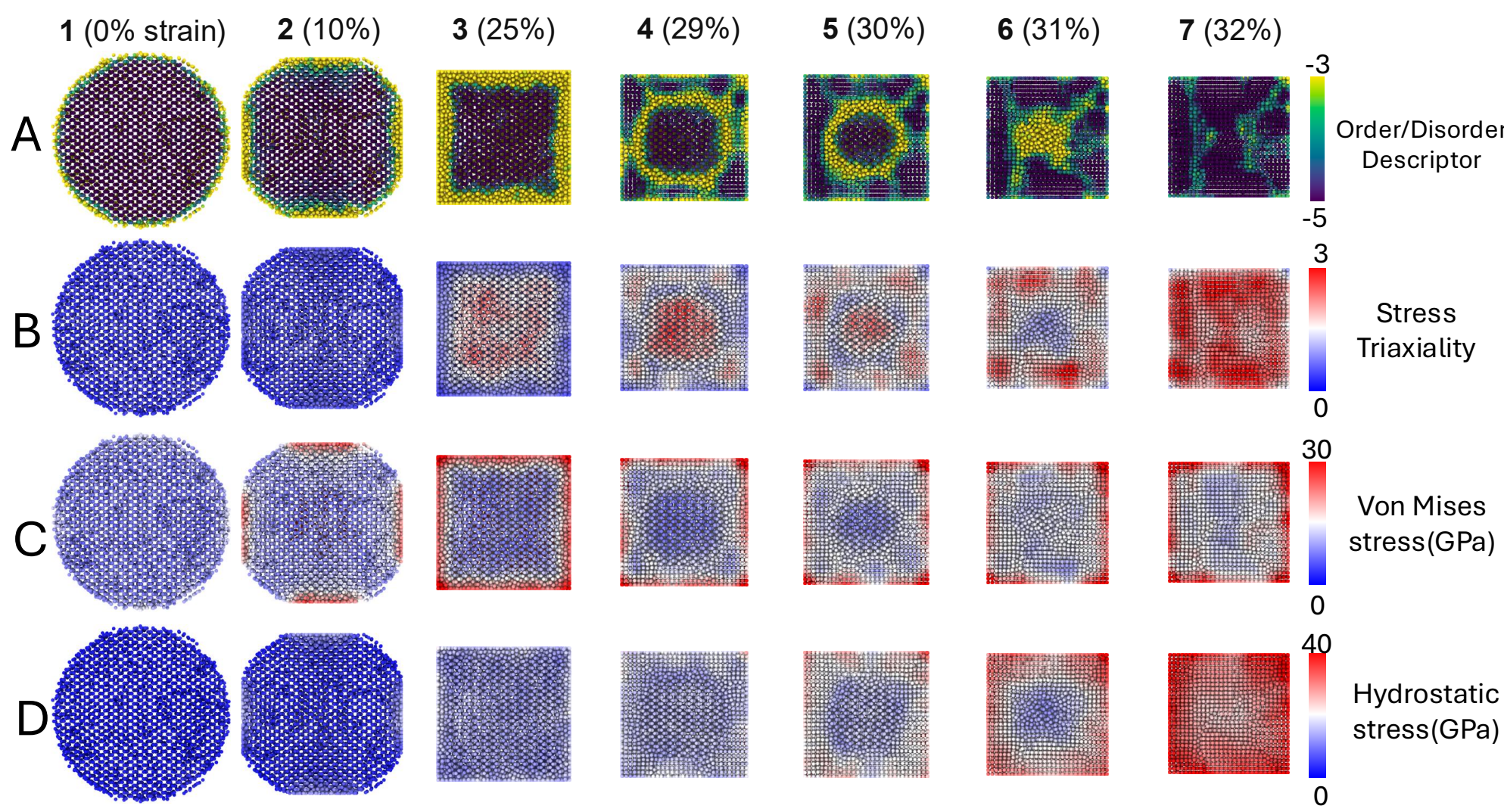

Figure S10: Cross-sectional snapshots of a 10-nm Si nanoparticle with its z axis aligned with [110]. The rows show (**A**) order/disorder descriptor maps, (**B**) stress-triaxiality fields, (**C**) von Mises stress fields, and (**D**) hydrostatic stress fields.

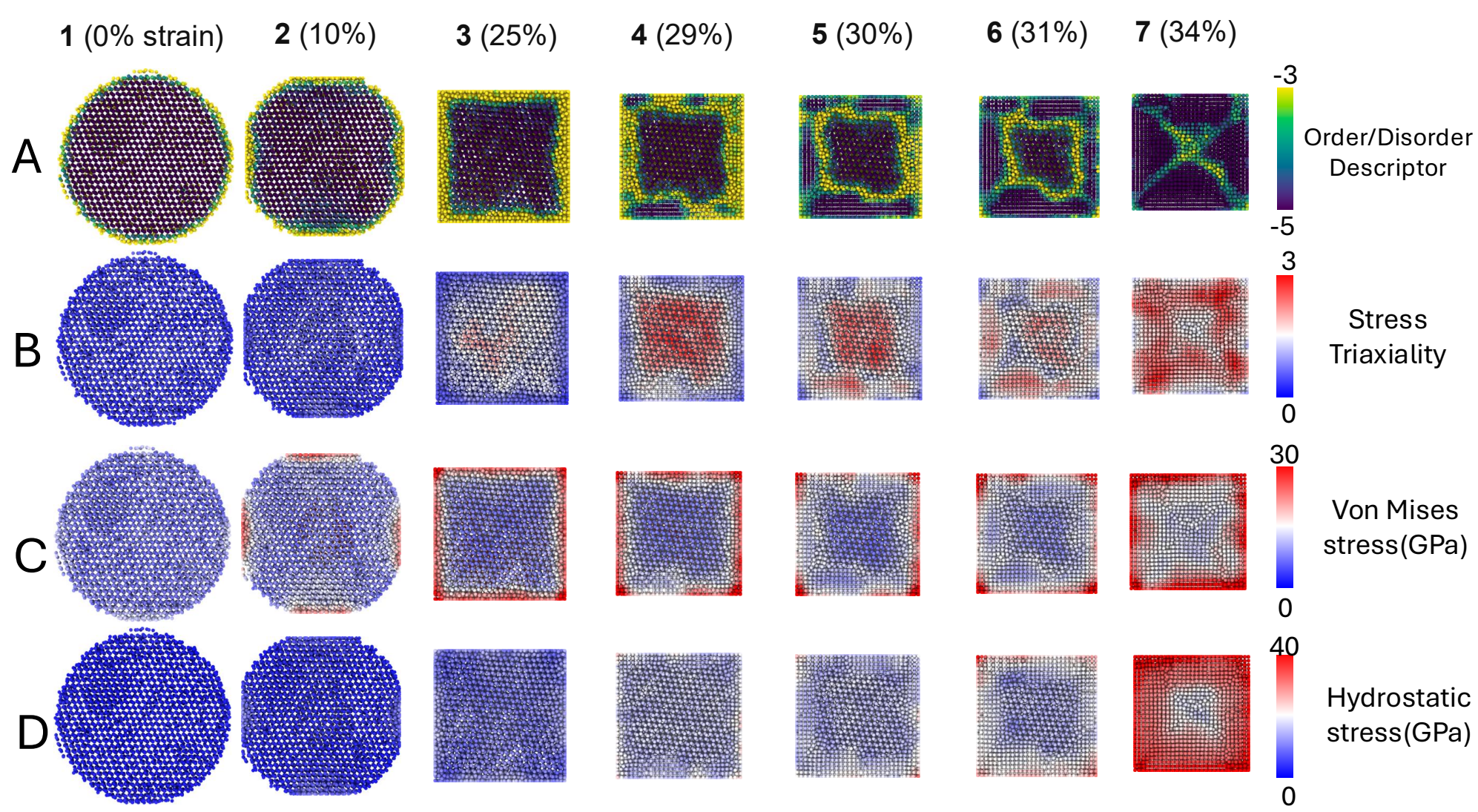

Figure S11: Cross-sectional snapshots of a 10-nm Si nanoparticle with its z axis aligned with [111]. The rows show (**A**) order/disorder descriptor maps, (**B**) stress-triaxiality fields, (**C**) von Mises stress fields, and (**D**) hydrostatic stress fields.

Taken together, these results show that, for the three orientations examined, crystallographic orientation shifts the onset of the transformation-associated plateau and modifies the detailed spatial distributions of disorder, ordering, and stress. However, it does not alter the qualitative sequence of mechanical and structural evolution.

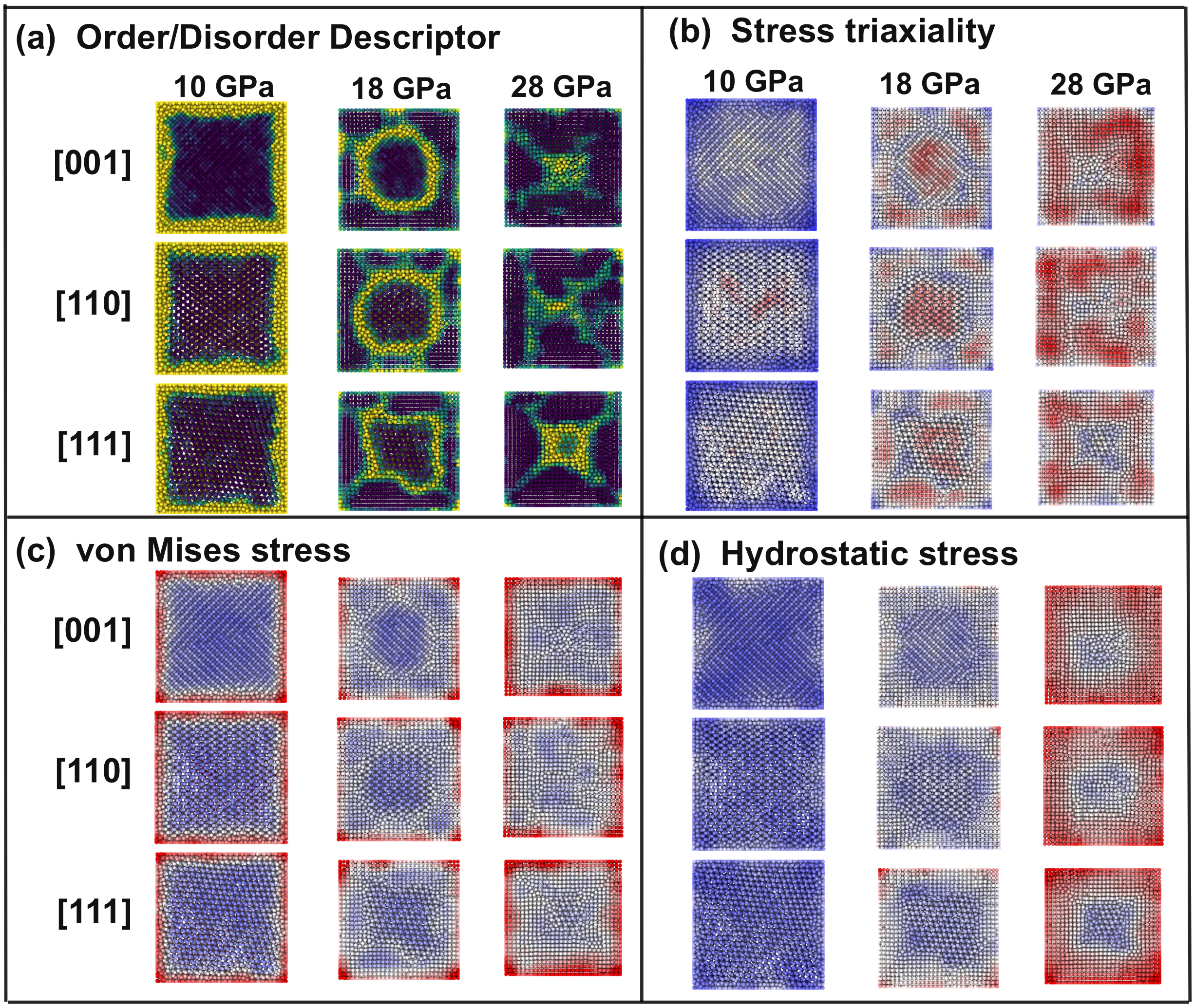

Figure S12: Cross-sectional snapshots of 10-nm Si nanoparticles with their z axes aligned with the [001], [110] and [111]. For each orientation, the columns show configurations selected at contact stresses of approximately 10, 18, and 28 GPa. The rows show (**a**) order/disorder descriptor, (**b**) stress triaxiality, (**c**) von Mises stress, and (**d**) hydrostatic stress.

Supplementary Note 4
Effects of Wall–Atom Interaction Stiffness K

The six planar indenters used in the single-particle model interact with the nanoparticle through a purely repulsive wall–atom interaction. The baseline simulations used an interaction stiffness of $K = 1000$ eV/Å^3. To evaluate the sensitivity of the simulated response to this parameter, we repeated the full loading simulation using $K = 100$ and 10 eV/Å^3. The particle orientation and loading protocol remain unchanged. Thus, only the wall–atom interaction stiffness was varied.

Reducing K shifts the transformation-associated response to higher imposed strains (Fig. S13). Both the onset of the plateau and the subsequent transition to hardening occur at progressively higher strains as K decreases. Nevertheless, all three curves retain the same mechanical sequence: an initial increase in contact stress, a transformation-associated plateau near 16 GPa, and subse-

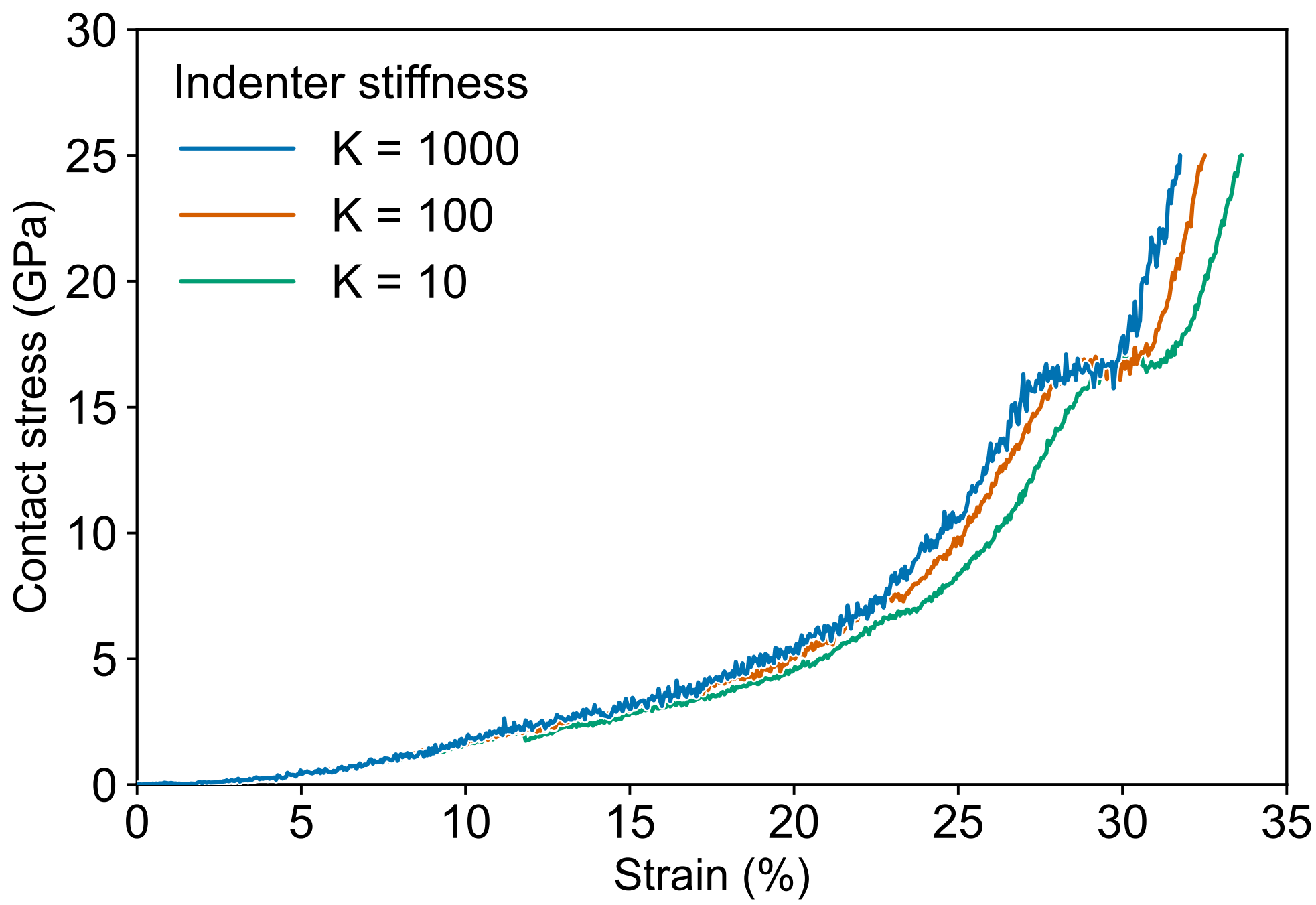

Figure S13: Contact stress–strain curves obtained from MD simulations of a 10-nm Si nanoparticle with its z axis aligned with the [001] crystallographic direction. The particle was compressed using wall–atom interaction stiffnesses of $K = 10$, 100, and 1000 eV/Å^3.

quent hardening. Thus, K primarily affects the relationship between imposed strain and contact stress rather than the characteristic plateau stress or the overall mechanical sequence.

Consistent with the mechanical response, the coordination-number and order/disorder descriptor population histories shift to higher imposed strains as K decreases while retaining the same overall sequence (Fig. S14). In all three cases, the loss of CN= 4 atoms is accompanied by a transient increase in the CN= 5 and CN= 6 populations, followed at later stages by increasing CN= 7 and CN= 8 populations. The order/disorder descriptor populations exhibit a corresponding shift along the strain axis. Differences observed at a fixed strain therefore primarily reflect the K-dependent timing of the transformation-associated response.

The strain-resolved central sections for $K = 100$ eV/Å^3 and $K = 10$ eV/Å^3 are shown in Figs. S15 and S16, respectively. In both cases, disordered material first develops near the particle surface and then extends inward. At larger compression, Si-V regions nucleate. The detailed distributions of stress triaxiality, von Mises stress, and hydrostatic stress vary with K, but the same broad surface-to-interior structural progression is observed.

Because a given imposed strain produces different contact stresses for different values of K, equal-strain snapshots do not necessarily represent comparable loading states. We therefore selected configurations from the three simulations at contact stresses of approximately 10, 18, and 28 GPa (Fig. S17). At matched contact stress, the simulations exhibit similar stages of structural evolution and broadly comparable spatial organization of mechanical properties, although local differences remain. This comparison supports the interpretation that the primary effect of K is to shift the imposed strain at which a given contact-stress regime is reached.

We further examined whether the observed structural patterns persist when continued indenter motion is stopped and the systems are allowed to relax. For each stiffness, configurations at a

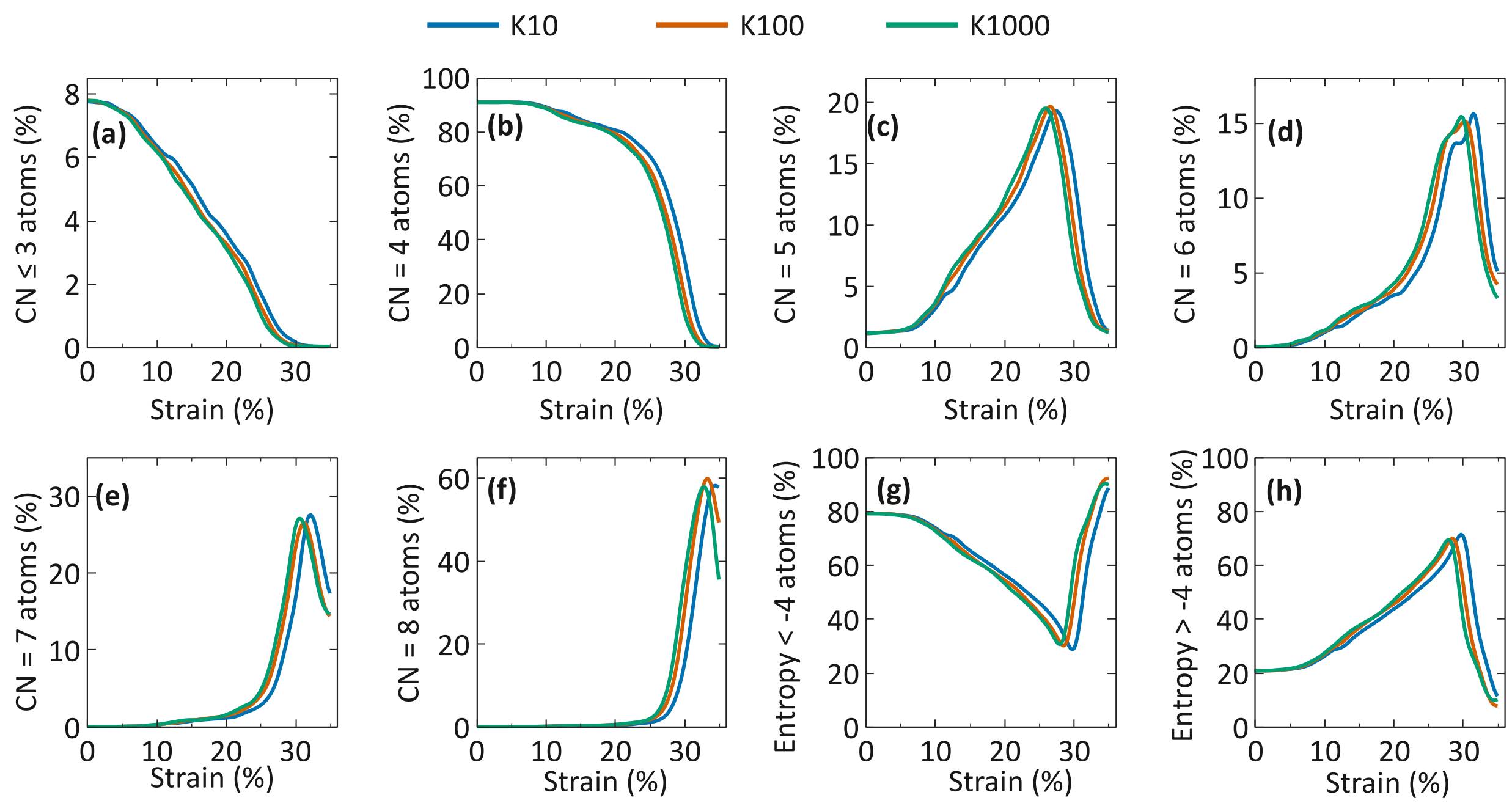

Figure S14: Global coordination-number and order/disorder descriptor populations as functions of strain for wall–atom interaction stiffnesses of $K = 10$, 100, and 1000 eV/Å^3. Panels (a–f) show the coordination-number populations calculated for all atoms using a 2.85 Å neighbor cutoff. Panels (g) and (h) show the order/disorder descriptor populations, respectively.

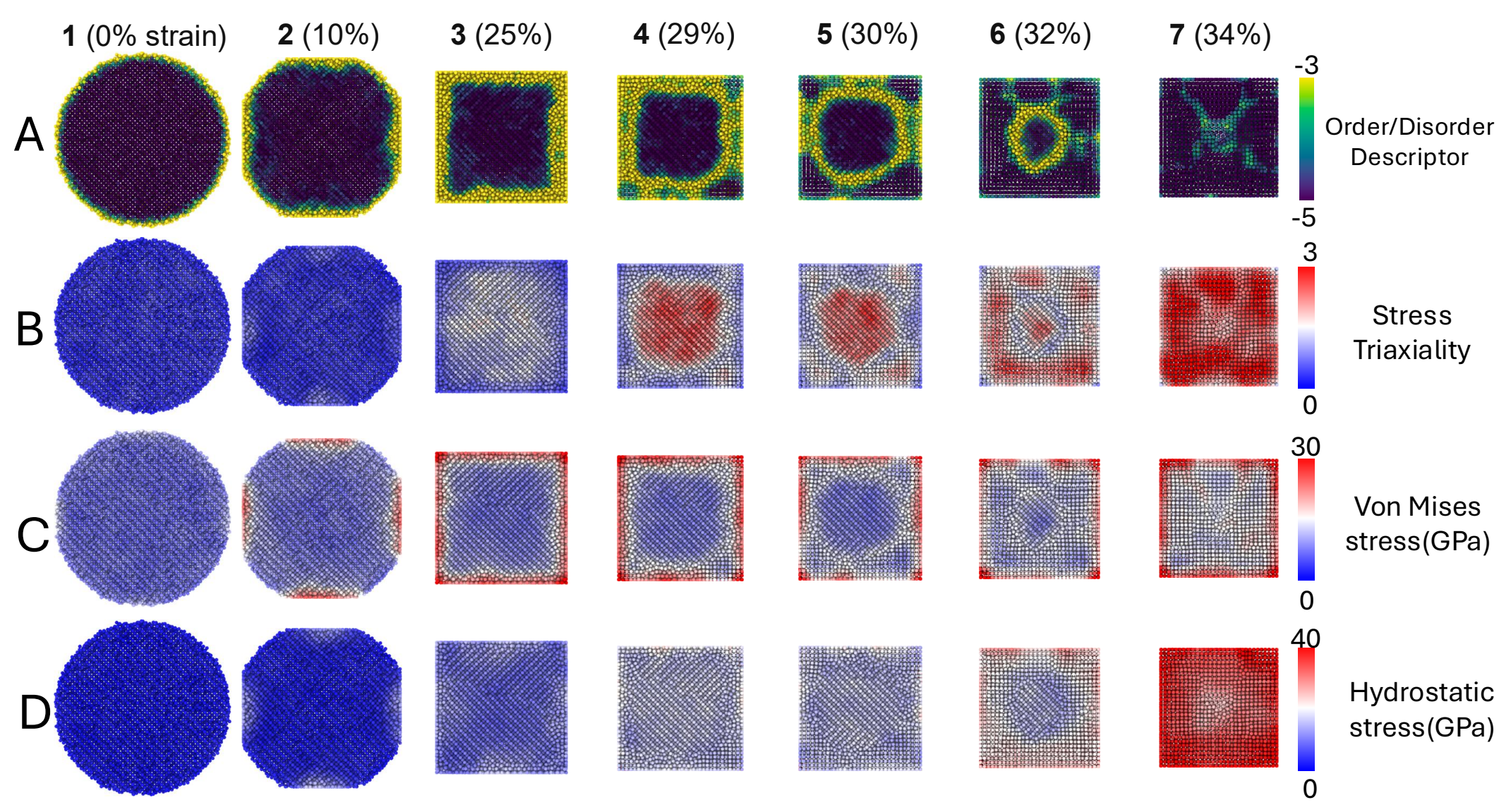

Figure S15: Cross-sectional snapshots of a 10-nm Si nanoparticle with its z axis aligned with the [001] under six-indenter compression using a wall–atom interaction stiffness of $K = 100$ eV/Å^3. The rows show (**A**) order/disorder descriptor, (**B**) stress triaxiality, (**C**) von Mises stress, and (**D**) hydrostatic stress.

comparable stage of Si-V nucleation were selected, after which the six indenter positions were held fixed and the system was allowed to relax. The heterogeneous order/disorder descriptor patterns

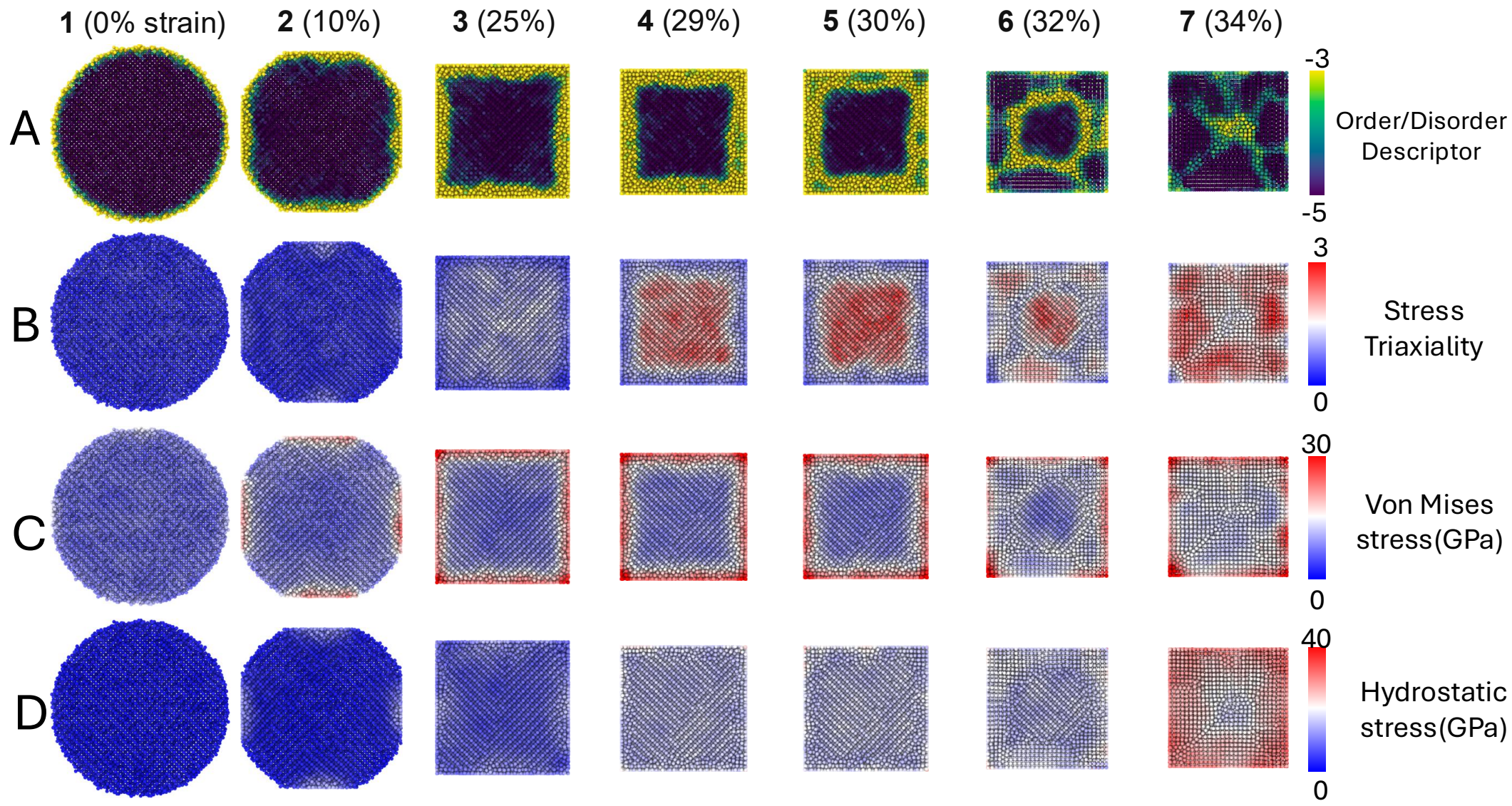

Figure S16: Cross-sectional snapshots of a 10-nm Si nanoparticle with its z axis aligned with the [001] under six-indenter compression using a wall–atom interaction stiffness of $K = 10$ eV/Å^3. The rows show (**A**) order/disorder descriptor, (**B**) stress triaxiality, (**C**) von Mises stress, and (**D**) hydrostatic stress.

associated with Si-V nucleation remain evident during the fixed-indenter relaxation, while the local stress fields redistribute (Fig. S18). This loading-hold test indicates that the observed structural heterogeneity is not solely a transient consequence of continued indenter motion.

Within the range $K = 10$–1000 eV/Å^3, the wall–atom interaction stiffness changes the imposed strain at which the transformation-associated features appear. However, it does not appreciably alter the characteristic plateau stress or the observed disorder-mediated Si-V formation pathway. The identification of this two-stage structural pathway is therefore robust to the choice of K within the range examined.

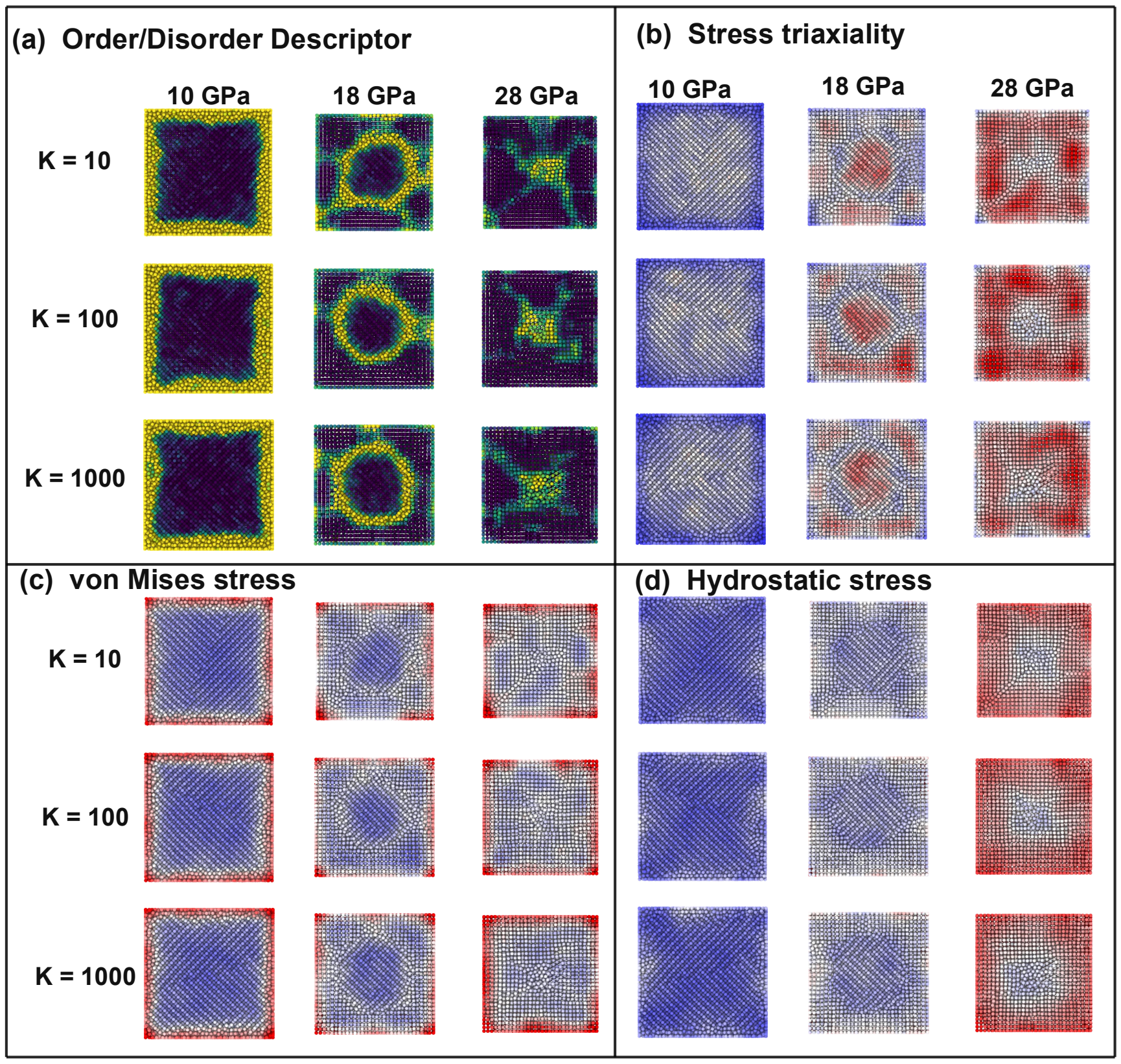

Figure S17: Cross-sectional snapshots of the 10-nm Si nanoparticle with its z axis aligned with the [001] crystallographic direction. The particle was compressed by six planar indenters using wall–atom interaction stiffnesses of K = 10, 100, and 1000 eV/Å^3. For each value of K, the columns show configurations selected at contact stresses of approximately 10, 18, and 28 GPa. The rows show (**a**) order/disorder descriptor, (**b**) stress triaxiality, (**c**) von Mises stress, and (**d**) hydrostatic stress.

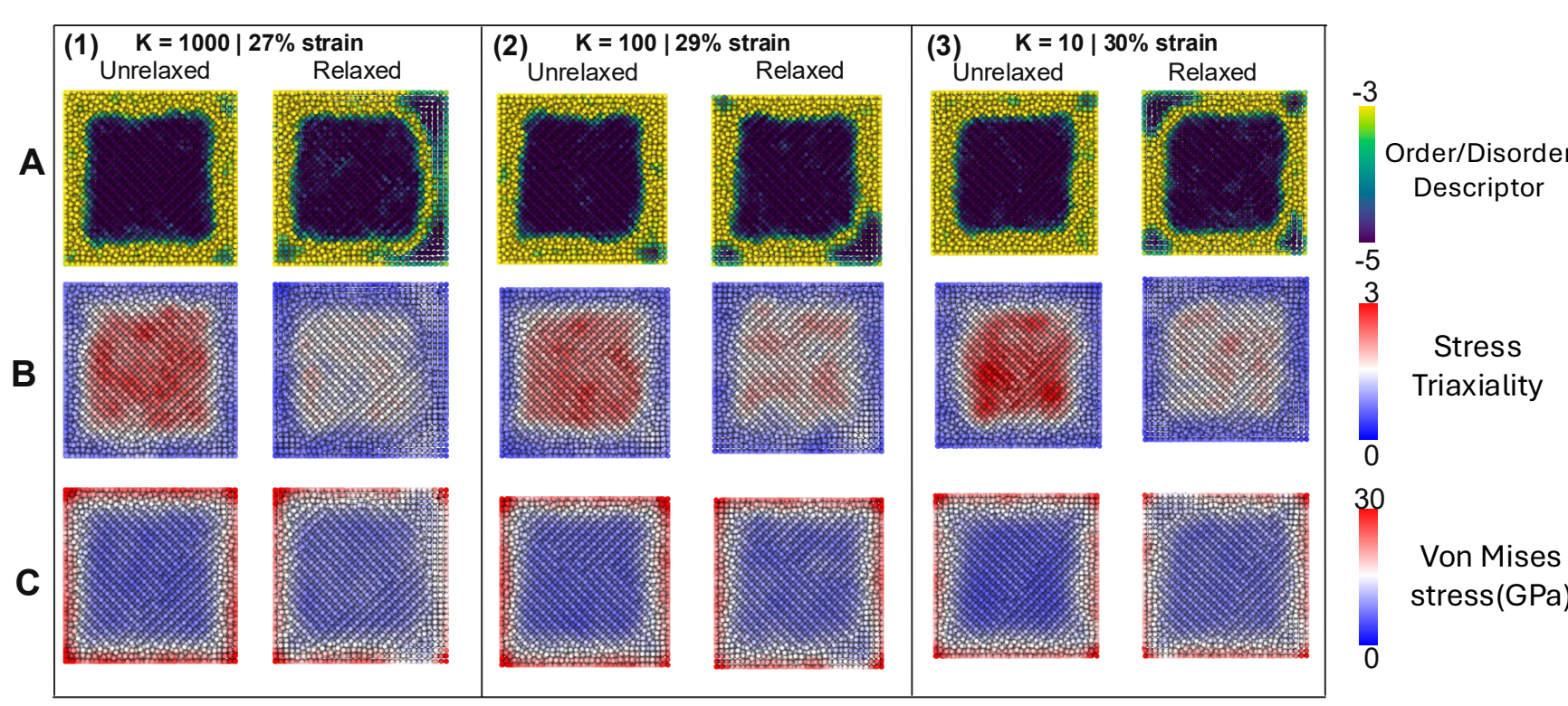

Figure S18: Comparison of configurations before and after MD relaxation with the six indenter positions held fixed. The three cases were selected at comparable stages of Si-V nucleation: (1) $K = 1000$ eV/Å^3 at 27% engineering strain, (2) $K = 100$ eV/Å^3 at 29% engineering strain, and (3) $K = 10$ eV/Å^3 at 30% engineering strain. The rows are colored according to (**A**) order/disorder descriptor, (**B**) stress triaxiality, and (**C**) von Mises stress.

Supplementary Note 5
Polymorphic Transitions during Unloading from the Si-V-rich State

The structural state analyzed here corresponds to point 2 on the first-unloading curve in Fig. 4(A) of the main text, where the nanoparticle has already undergone substantial unloading from the Si-V-rich compressed state but has not yet fully transformed into the final LDA-like amorphous state. At this stage, the structure is heterogeneous and contains coexisting amorphous regions, remnant Si-V-like ordered regions, and localized Si-II-like ordered regions. To examine the stability of these remnant ordered regions under the unloading stress state, we performed a fixed-indenter relaxation simulation at 300 K using the same GAP potential and thermostat, with all six indenter positions held fixed. Thus, no additional macroscopic unloading strain was imposed, and the observed structural evolution reflects local relaxation under fixed triaxial confinement.

As shown in Fig. S19(A–C), the unloading structure does not undergo a uniform reverse transformation from Si-V to amorphous Si. Before relaxation, the order/disorder descriptor map shows disordered regions adjacent to remnant ordered domains (Fig. S19(A)). After fixed-indenter relaxation, these disordered regions expand and the ordered–disordered interfaces evolve, indicating that structural relaxation proceeds preferentially near pre-existing amorphous regions. The phase map further shows that the remaining ordered material contains both Si-V-like and Si-II-like ordered regions rather than a single crystalline phase (Fig. S19B). The coordination-number map indicates that this relaxation is accompanied by a reduction in local coordination from the high-density crystalline phase toward lower-coordinated amorphous configurations (Fig. S19C). The RDF and ADF analyses further validate the local phase assignments. The atoms classified as Si-II-like exhibit radial and angular peak positions closer to the Si-II reference than to the Si-V reference (Fig. S19D,E).

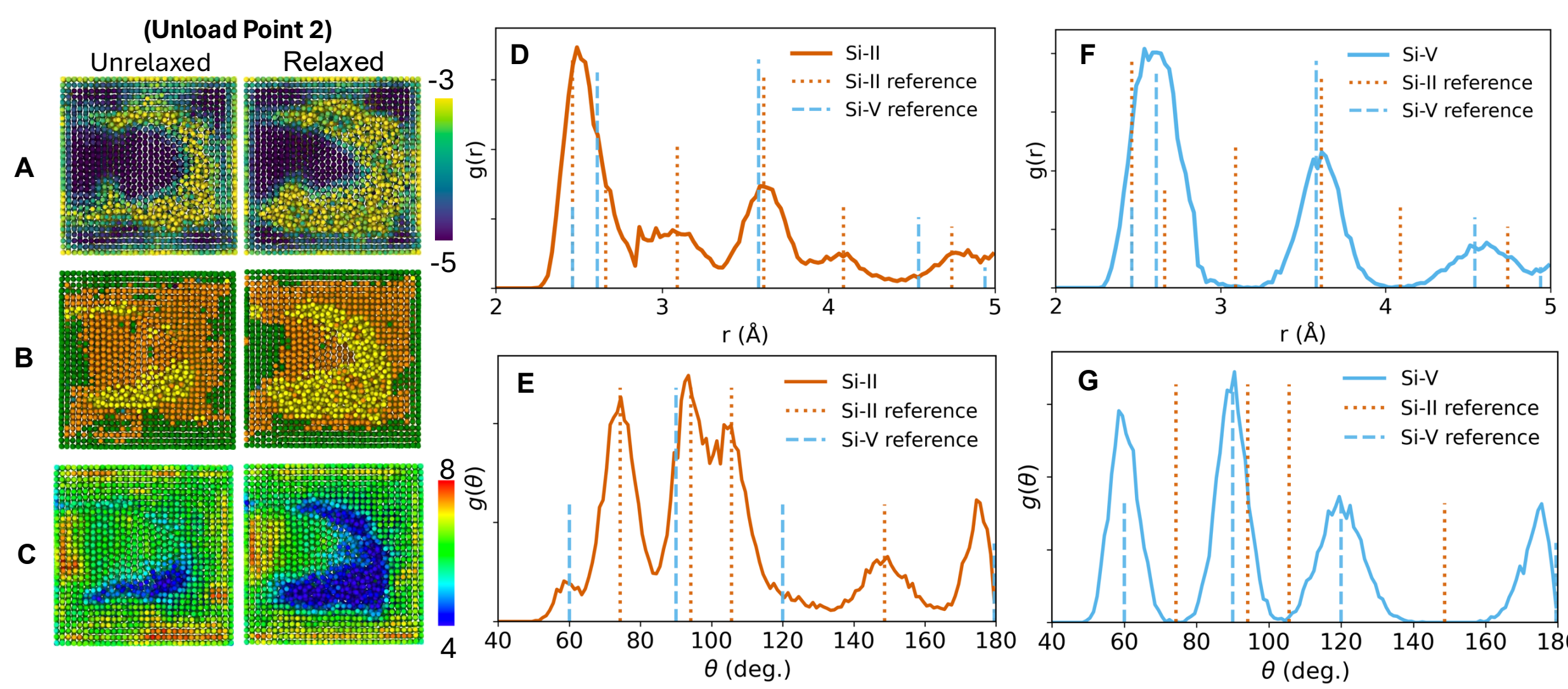

Figure S19: Fixed-indenter relaxation analysis of polymorphic relaxation during the first unloading from the Si-V-rich state. The configuration corresponds to point 2 on the first-unloading curve in the main text. (**A**) Cross-sectional snapshots colored by order/disorder descriptor before and after fixed-indenter relaxation. (**B**) Corresponding phase maps. (**C**) Coordination number maps before and after relaxation. (**D**,**E**) RDF and ADF of the Si-II-like ordered region, compared with Si-II and Si-V reference peak positions. (**F**,**G**) RDF and ADF of the Si-V-like ordered region, compared with Si-II and Si-V reference peak positions.

In contrast, the Si-V-like subset retains RDF and ADF signatures that are more consistent with the Si-V reference (Fig. S19F,G). Because these Si-II-like regions are finite, localized, and embedded within a mixed amorphous/crystalline environment, we describe them as localized Si-II-like order rather than fully developed bulk Si-II crystals. The coexistence of Si-II-like and Si-V-like ordered regions during unloading suggests that these local structures are locally accessible under the non-hydrostatic unloading stress state and consistent with previous GAP-based simulations of stress-assisted phase transformations [S2].

These results support the unloading mechanism described in the main text. During the first unloading branch, the Si-V-rich nanoparticle relaxes heterogeneously through two coupled routes: disordering of Si-V-like ordered regions into amorphous Si and local transformation of remnant crystalline regions toward Si-II-like order. The formation of Si-II-like order reflects a density reduction of crystalline regions relative to the highly compressed high-density crystalline phase. Continued amorphization during unloading is accompanied by volume expansion, which contributes to the transformation back-stress observed in the unloading stress–strain curve. After complete unloading, the remnant ordered regions disappear and the nanoparticle relaxes into an LDA-like amorphous state with a predominantly fourfold-coordinated network. Importantly, unloading generates a structural pathway distinct from the first-loading process discussed in Supplementary Note 2: instead of the Si-I$\rightarrow$HDA/VHDA-like a-Si$\rightarrow$Si-V pathway, the unloading pathway proceeds through heterogeneous relaxation of the high-density crystalline phase, involving both amorphization and local evolution toward Si-II-like order.

Supplementary Note 6
Transformation Pathway in a 5-nm Si Nanoparticle

To assess whether the transformation sequence observed in the 10-nm particle persists at a smaller size, we simulated a 5-nm diameter Si nanoparticle containing 3,265 atoms with the z-axis aligned along [001]. The same surface-preparation procedure and absolute indenter velocity were used. Because this produces a higher nominal strain rate for the smaller particle, the calculation is treated as a qualitative size comparison. As shown in Fig. S20, the 5nm particle follows the same two-step Si-I→a-Si→Si-V sequence observed in the 10nm particle. The amorphous region first expands inward from the particle surface and contact regions, progressively consuming the Si-I core. Si-V subsequently nucleates and grows during further compression. Compared with the 10-nm particle, the 5-nm particle retains a larger fraction of amorphous Si between the Si-V domains, consistent with the stronger influence of the free surface. Nevertheless, the transformation sequence remains unchanged. Thus, the amorphization-mediated pathway persists in the 5nm Si nanoparticle.

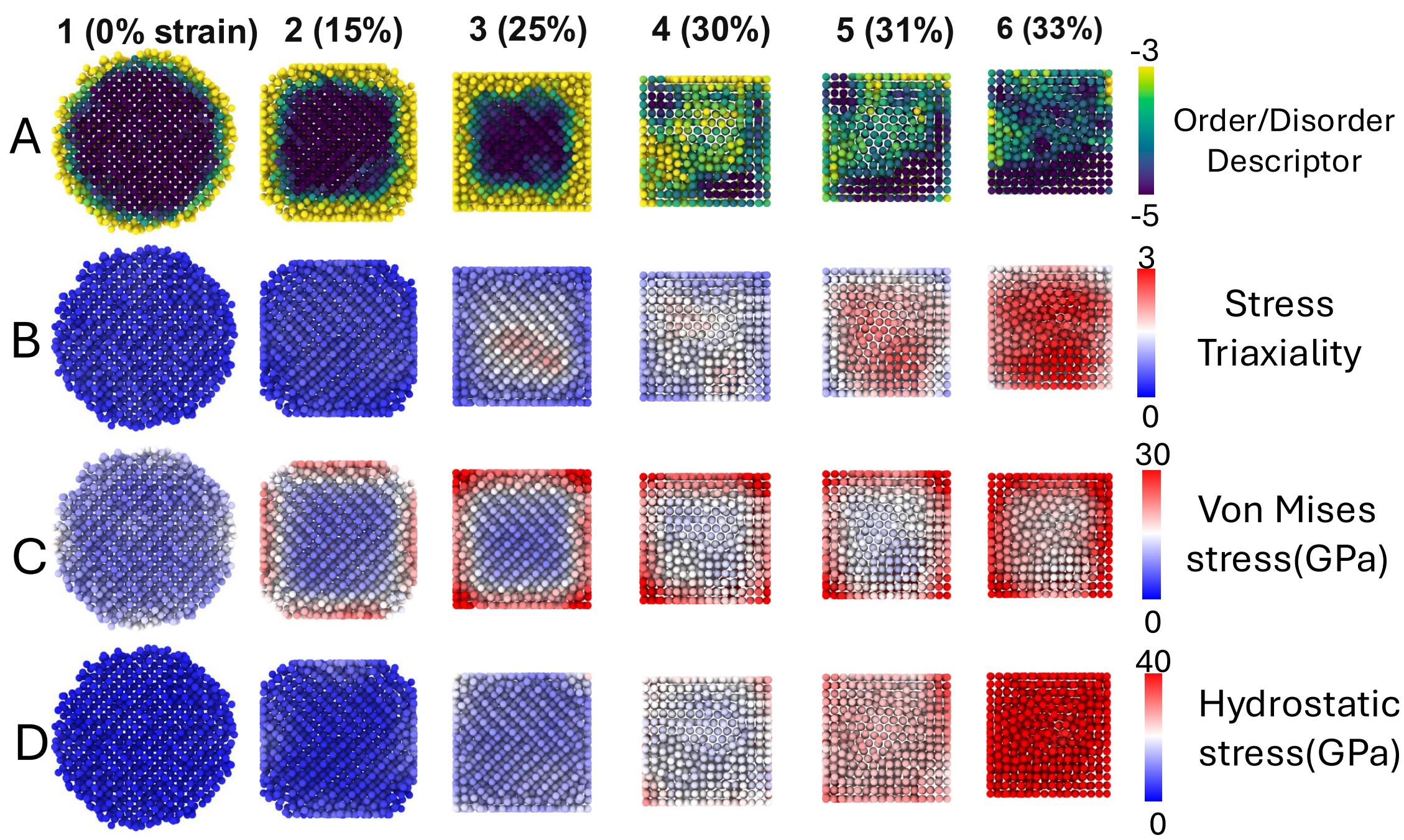

Figure S20: Cross-sectional snapshots of a 5-nm Si nanoparticle under triaxial compression, displayed as a function of engineering strain (columns). The rows show (**A**) order/disorder descriptor, (**B**) phase classification, (**C**) stress triaxiality, (**D**) von Mises stress, and (**E**) hydrostatic stress.

Supplementary Note 7
Initial Surface Structure Analysis

Because amorphization initiates from the particle surface, we characterized the initial surface structure before compression. The surface region was defined as the outermost 5 Å shell measured inward from the OVITO alpha-shape surface [S5]. The order/disorder descriptor distributions for the three orientations show similar distributions(Fig. S21). Each distribution contains a diamond-related population together with intermediate- and surface environments. Thus, the annealing and cooling

procedure produces a partially reconstructed and disordered surface while preserving substantial diamond-related order.

We also repeated the thermal preparation using annealing temperatures of 800 and 1000 K. The resulting order/disorder descriptor distributions are also nearly identical (Fig. S22), indicating that the initial surface structure is only weakly affected by annealing temperature over our tested range.

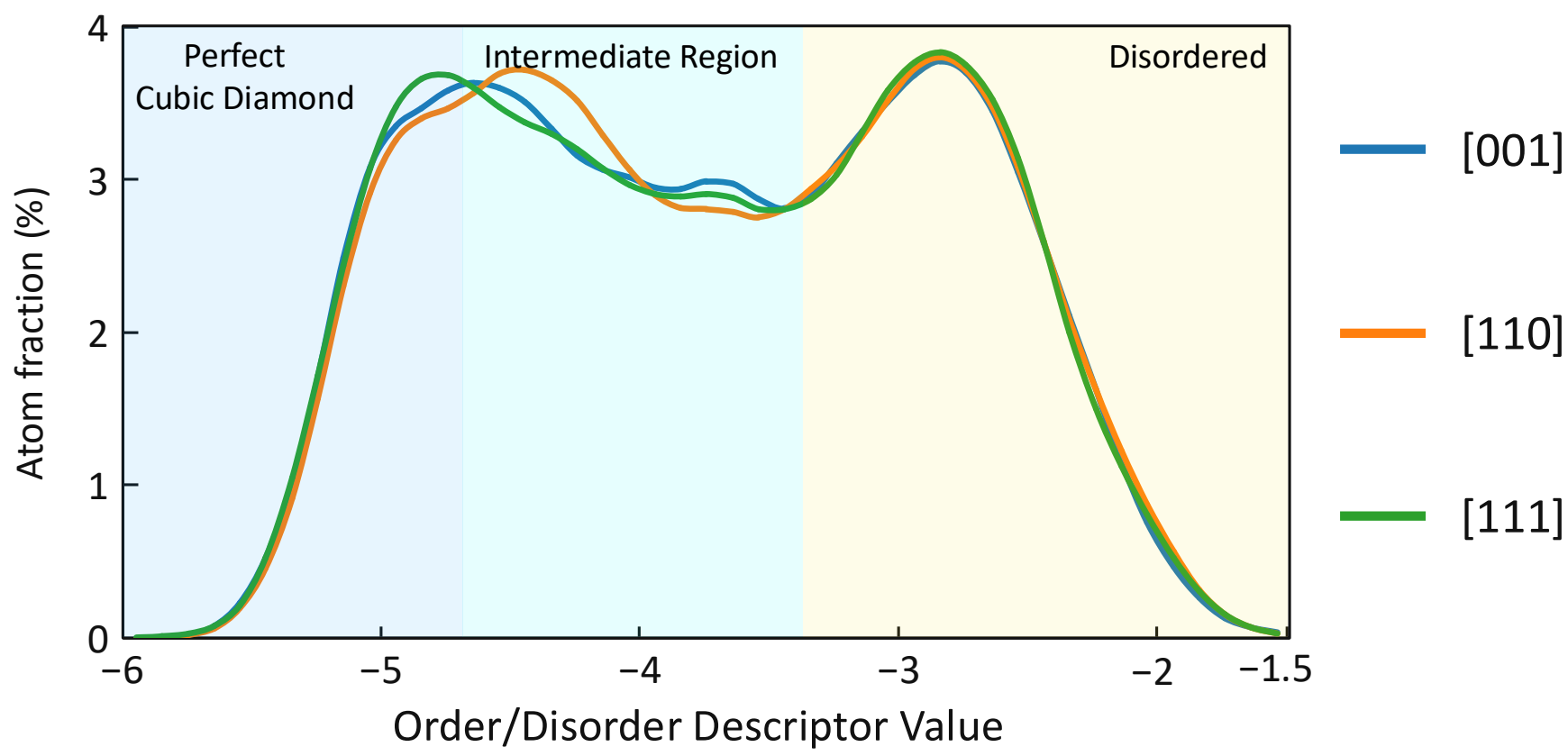

Figure S21: Order/disorder descriptor distributions within the outermost 5 Å shell after thermal treatment for different orientations listed in Table S1.

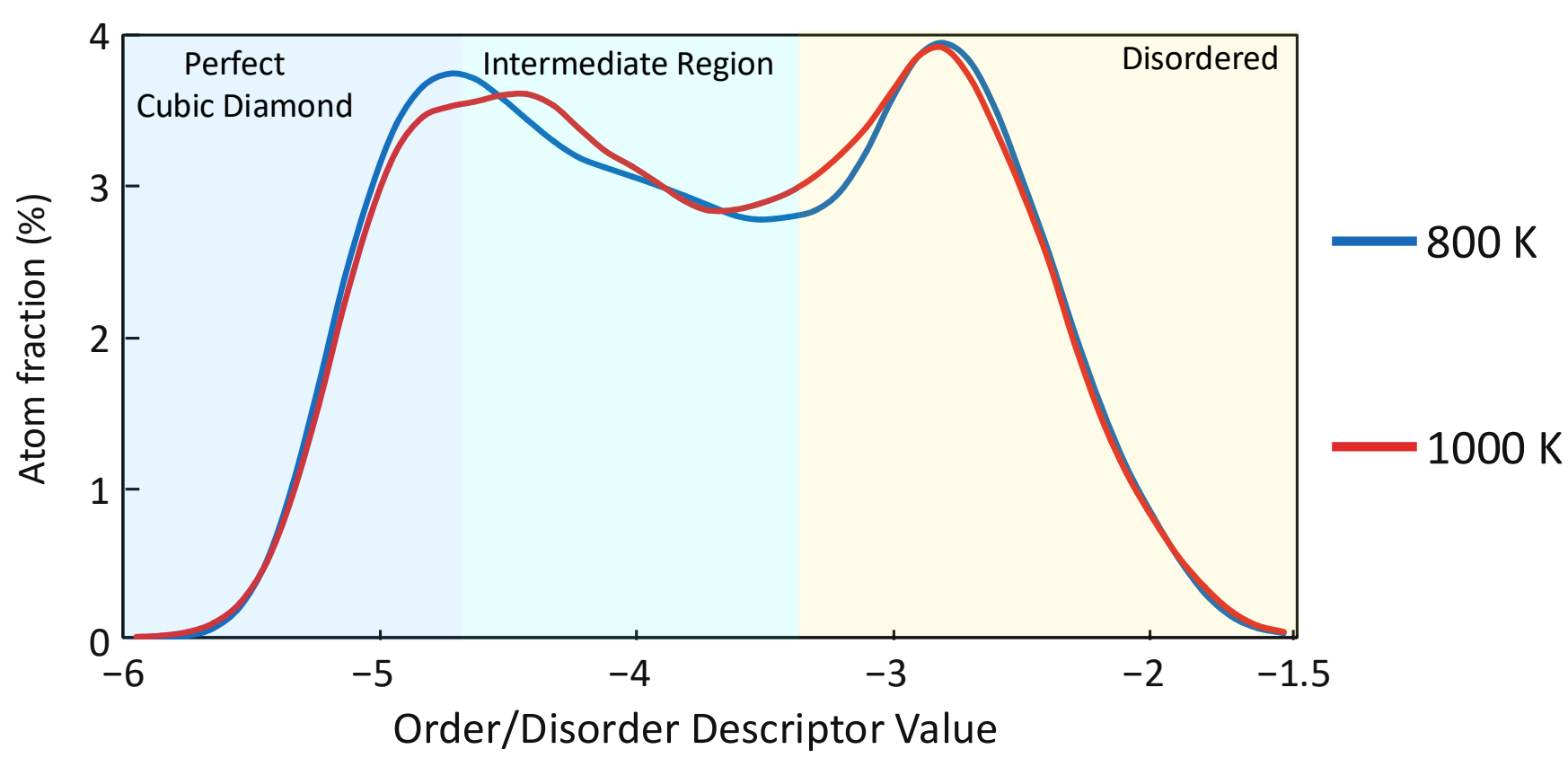

Figure S22: Order/disorder descriptor distributions of atoms within the outermost 5 Å shell after thermal annealing at 800 and 1000 K.

References

[S1] D. Chrobak, N. Tymiak, A. Beaber, O. Ugurlu, W. W. Gerberich, and R. Nowak. Deconfinement leads to changes in the nanoscale plasticity of silicon. *Nat. Nanotechnol.*, 6:480–484, 2011.

[S2] G. J. Ge, F. Rovaris, D. Lanzoni, L. Barbisan, X. B. Tang, L. Miglio, A. Marzegalli, E. Scalise, and F. Montalenti. Silicon phase transitions in nanoindentation: Advanced molecular dynamics simulations with machine learning phase recognition. *Acta Mater.*, 263:119465, 2024.

[S3] Y. Hong, N. Zhang, and M. A. Zaeem. Metastable phase transformation and deformation twinning induced hardening-stiffening mechanism in compression of silicon nanoparticles. *Acta Mater.*, 145:8–18, 2018.

[S4] Emile Maras, Oleg Trushin, Alexander Stukowski, Tapio Ala-Nissila, and Hannes Jónsson. Global transition path search for dislocation formation in ge on si (001). *Computer Physics Communications*, 205:13–21, 2016.

[S5] Alexander Stukowski. Computational analysis methods in atomistic modeling of crystals. *JOM*, 66(3):399–407, 2014.

[S6] P. Valentini, W. W. Gerberich, and T. Dumitrică. Phase-transition plasticity response in uniaxially compressed silicon nanospheres. *Phys. Rev. Lett.*, 99:175701, 2007.
